# Research trends on the quality of life in patients with metabolic dysfunction-associated fatty liver diseases: a scientific metrology study

**DOI:** 10.3389/fnut.2025.1689280

**Published:** 2025-10-10

**Authors:** Can Huang, Meng Chen, Yanfang Sun, Lin Zhang, Wei Liu

**Affiliations:** Department of Pharmacy, Beijing You'an Hospital Affiliated to Capital Medical University, Beijing, China

**Keywords:** bibliometric analysis, quality of life, MAFLD, metabolic comorbidities, research trends

## Abstract

**Background:**

Metabolic dysfunction-associated fatty liver disease (MAFLD) has become the most prevalent chronic liver disease globally, significantly impairing patients’ quality of life (QOL) through complex interactions with metabolic comorbidities, psychological factors, and disease progression. Despite growing research interest, a comprehensive bibliometric analysis of QOL studies in MAFLD remains lacking.

**Methods:**

Publications focusing on MAFLD and QOL were retrieved from the Web of Science Core Collection and Scopus. Bibliometric data, including titles, authors, institutions, countries, keywords, and citations, were extracted and analyzed using Bibliometrix (R-package) and VOSviewer. Annual publication trends, geographical contributions, institutional collaborations, journal influence, author networks, and keyword evolution were visualized.

**Results:**

A total of 1,347 publications (2004–2025) were included, with an annual growth rate of 18.76%. The United States (*n* = 368, 27.3%) was the most productive country, followed by China (*n* = 171, 12.7%) and Italy (*n* = 94, 7.0%). Leading institutions included the University of California System (*n* = 67) and Harvard University (*n* = 54). Key journals included Hepatology (*n* = 27) and Journal of Hepatology (*n* = 15). Keyword analysis identified five clusters: obesity management, metabolic health, MAFLD pathology, health impacts, and risk factors. Emerging trends focused on drug therapy, lifestyle interventions, and psychosocial support.

**Conclusion:**

Research on MAFLD patients’ QOL is growing fast. Effective management needs integrated strategies (lifestyle, psychological support, multidisciplinary management, precision pharmacology) tailored to individuals. Future research should prioritize long-term data, clarity of mechanisms, and equitable interventions for patients’ holistic well-being.

## Introduction

Metabolic dysfunction-associated fatty liver disease (MAFLD), previously termed non-alcoholic fatty liver disease (NAFLD), has emerged as the most prevalent chronic liver disease worldwide, affecting approximately one-third of the global adult population (1–3). The disease spectrum encompasses metabolic dysfunction-associated fatty liver (MAFL), metabolic dysfunction-associated steatohepatitis (MASH), and progression to liver fibrosis, cirrhosis, and hepatocellular carcinoma (HCC) (4, 5). Beyond hepatic impairment, MAFLD is closely linked to metabolic syndrome (MetS), type 2 diabetes mellitus (T2DM), cardiovascular disease (CVD), and chronic kidney disease (CKD), forming a complex network of comorbidities that exacerbate patient burden (6, 7).

Quality of life (QOL) refers to a multidimensional construct that includes physical function, emotional well-being, social participation, and disease-related symptom burden. Common assessment tools include the Short Form 36 Health Survey (SF-36, which evaluates 8 domains such as physical role and mental health), the Chronic Liver Disease Questionnaire (CLDQ, specific to liver diseases, focusing on abdominal symptoms and fatigue), and the Patient-Reported Outcomes Measurement Information System (PROMIS, which measures global QOL with high sensitivity to metabolic comorbidities). Notably, MAFLD significantly impairs QOL, with patients reporting reduced physical function, increased fatigue, emotional distress, and social withdrawal (8). Studies comparing MAFLD patients to the general population consistently demonstrate lower scores in domains such as abdominal symptoms, activity levels, and anxiety (9–11). Despite this, research on QOL in MAFLD remains fragmented, with limited synthesis of global trends, collaborative networks, or emerging priorities.

Bibliometric analysis offers a quantitative framework to map the intellectual structure of a field, identifying key contributors, influential works, and knowledge gaps (12, 13). To the best of our knowledge, no bibliometric analysis focusing on the QOL in patients with MAFLD has been conducted. This study aims to conduct a comprehensive bibliometric analysis of publications on MAFLD patients’ QOL, with objectives to: (1) characterize publication trends over time; (2) identify leading countries, institutions, and researchers; (3) visualize collaborative networks; (4) analyze keyword clusters to reveal research hotspots; and (5) highlight emerging frontiers to guide future research.

## Methods

### Search strategy

In May 2025, electronic literature searches were performed via two databases: the Web of Science Core Collection (WoSCC) and Scopus. WoSCC is a large-scale database that includes bibliometric details for each entry and is widely utilized in bibliometric research (14, 15). It was chosen as one of the data sources given its status as a high-quality academic literature repository, which researchers broadly recognize as a favorable option for bibliometric analyses.

The retrieval strategy combined terms related to MAFLD and quality of life: (Topic: (“MASLD” OR “metabolic dysfunction-associated steatotic liver disease” OR “MAFLD” OR “metabolic dysfunction-associated fatty liver disease” OR “NAFLD” OR “nonalcoholic fatty liver disease”)) AND (Topic: (“quality of life” OR “health-related quality of life” OR “HRQoL” OR “QOL” OR “life quality” OR “patient-reported outcomes”)).

Scopus is also a prominent academic database widely used in research. For the Scopus database search, the same retrieval logic regarding the content of search terms was applied.

**Figure 1:**
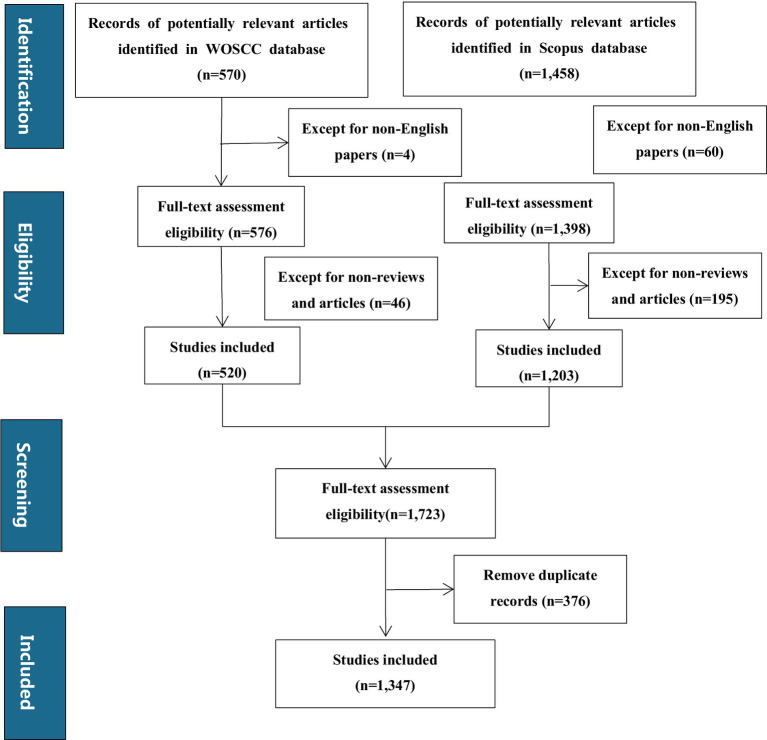
Flow diagram for the screening procedure.

After retrieving records from both WoSCC and Scopus databases, the subsequent steps of eligibility assessment, screening for non-English papers, non-reviews and articles, and removing duplicate records were carried out as shown in the flowchart (Figure 1).

### Data extraction

Full records and cited references of the included publications were exported in tab-delimited text format. Extracted bibliometric parameters encompassed titles, abstracts, keywords, authors, affiliations, countries/regions, publication year, journal names, and references. All data underwent double-verification to ensure accuracy; any inconsistencies were resolved by re-examining the original articles.

Keywords were consolidated using multiple criteria: semantic equivalence morphological variations abbreviations and their full forms domain-specific terminological standards and contextual links (where terms frequently co-occur and are closely related). These criteria were adopted to ensure that terms representing the same core concept were merged thereby improving the replicability and clarity of the analysis.

### Data analysis

This study used the Bibliometrix package in R software (version 4.4.3) and VOSviewer (version 1.6.20) for bibliometric analysis and the creation of scientific knowledge maps (16, 17). The Bibliometrix R package was mainly used for analyzing annual production, country-wise production, authors’ contributions over time, local impacts of sources based on the *H* index, and trending topics (18, 19). VOSviewer, a powerful bibliometric tool, was used to create knowledge maps based on web data and to visualize and explore these maps (20). This study leveraged VOSviewer for clustering analyses of countries, institutions, journals, authors, citations, and keywords due to its intuitive and clear performance in clustering tasks (21). Both tools analyze co-occurrence (e.g., keywords appearing together in publications) to map relationships and identify natural clusters of tightly linked items. VOSviewer primarily visualizes these networks and clusters directly from co-occurrence data, using distance and color, while Bibliometrix calculates the underlying matrices, offers statistical clustering methods, and detects trends through features like thematic evolution diagrams over time.

## Results

### Literature acquisition and time-related trends

Bibliometric landscape overview: A total of 1,347 documents (Figure 1) were identified through searches on the Web of Science platform and Scopus. Most of these retrieved publications (1,025 articles, accounting for 76% of the total) were issued between 2018 and 2025; a small number of earlier journal articles (322 articles, accounting for 24% of the total) were included, with publication years ranging from 2004 to 2017. Among the literature from 2004 to 2017, 2017 was the first year with more than 50 publications. For subsequent analyses, a timeline-driven method was adopted, with the study period set as 2004–2025. The retrieval outcomes encompassed 617 journals, with the annual growth rate of published articles reaching 18.76%. Each document was cited an average of 54.8 times, and the total number of contributing authors stood at 7,607 (Table 1).

**Table 1:** Synopsis of literature search outcomes.

Account	Results
Main information about data	
Timespan	2004:2025
Sources (Journals, Books, etc.)	617
Documents	1,347
Annual Growth Rate %	18.76
Document Average Age	4.99
Average citations per doc	54.8
Document contents	
Keywords Plus (ID)	10,186
Author’s Keywords (DE)	2,684
Authors	
Authors	7,607
Authors of single-authored docs	74
Document types	
Article	741
Review	606

**Figure 2:**
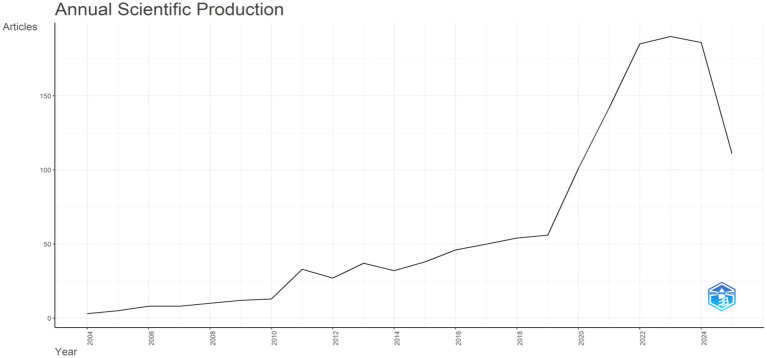
Distribution of yearly article outputs from 2004 to 2025.

Publication dynamics: Figure 2 illustrates the annual scientific output (measured by the number of articles) from 2004 to 2025. In the early phase (2004–2010), output remained low and grew slowly. From the early 2010s onward, it gradually showed an upward trend with minor fluctuations. Around 2018, growth began to accelerate sharply, leading to a rapid rise. A prominent peak emerged around 2022 thereafter, output has fluctuated at a relatively high level.

### Geographical and institutional output

**Figure 3:**
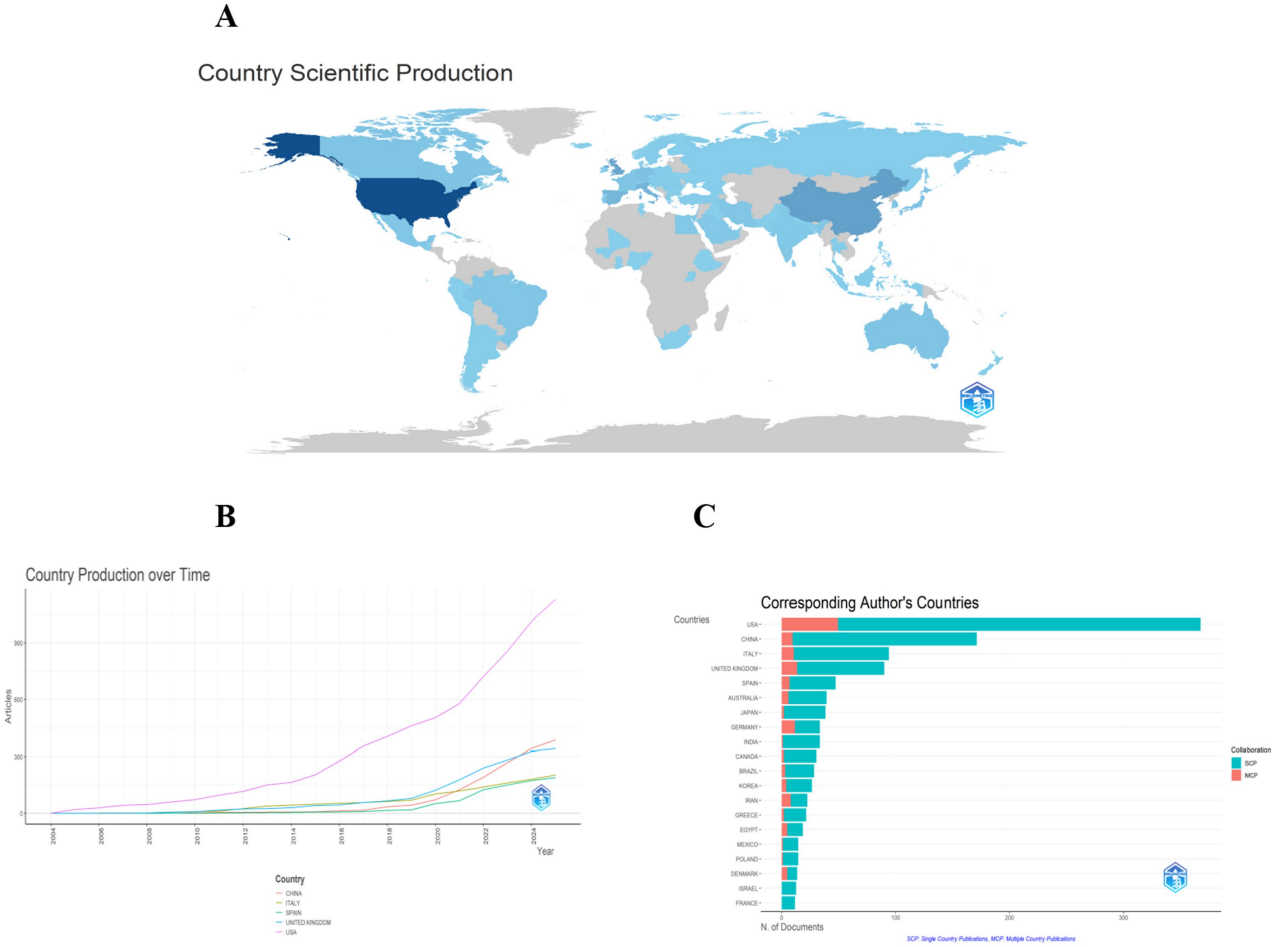
Panel **(A)** is a geographical distribution map of national research output, where dark blue areas (such as the United States) represent countries with high output. Panel **(B)** shows the trend of literature publication volume in major countries from 2004 to 2025. It can be seen that the United States has experienced a significantly higher growth rate in literature output than other countries since 2018, while China has shown rapid growth after 2020. Panel **(C)** compares the proportion of single-country published literature (SCP) and multi-country collaborative literature (MCP) among various countries.

**Figure 4:**
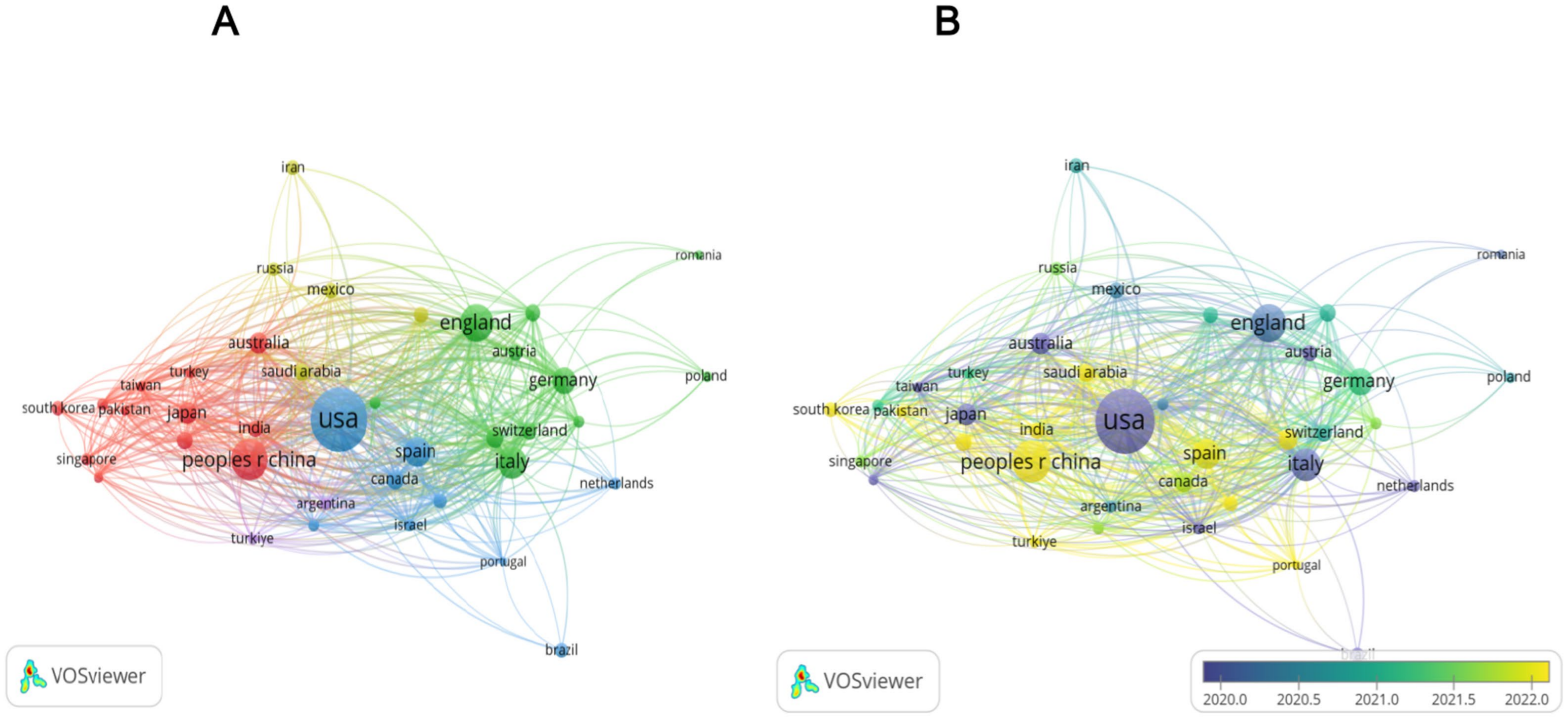
Analysis of countries involved in research on the QOL of MAFLD Patients. Panel **(A)** is a cluster diagram of the national cooperation network. In this diagram, different colors denote 5 cooperation clusters. For example, the United States, the United Kingdom, and Canada form the red cluster. The size of the nodes is positively correlated with the volume of literature output, and the thickness of the lines between nodes represents the intensity of cooperation. Panel **(B)** is a distribution map of the average publication year of literature by country. Blue nodes, like those of Italy, represent countries that were active in the early stage (before 2015). Meanwhile, yellow nodes, such as those of China, represent countries that have been active in the recent stage (after 2020).

National productivity and collaboration: The national publication output analysis showed that 77 countries/regions contributed to the field. The country-wise distribution of publications is presented in Table 2 and Figure 3. The United States (*n* = 368) was the most prolific nation, accounting for 27.3% of all publications, followed by China (*n* = 171, 12.7%), Italy (*n* = 94, 7%), United Kingdom (*n* = 90, 6.7%), Spain (*n* = 47, 3.5%), and Australia (*n* = 39, 2.9%). To present the international collaborative network, we utilized the co-authorship-country module in VOSviewer (Figure 4). A total of 39 prolific countries/regions (with at least 5 publications) formed a cooperative network. Among these, the United States, the United Kingdom, China, Spain, and Italy emerged as large nodes with relatively thick links. The United States had the highest total link strength (TLS = 264) in cooperation and collaborated with 36 prolific countries. Among them, China and the United Kingdom showed close academic cooperation with the United States.

**Table 2:** The 10 most productive nations in research on the QOL of MAFLD patients.

Rank	Country	Articles	Articles %	SCP	MCP	MCP %
1	USA	368	27.3	319	49	13.3
2	CHINA	171	12.7	162	9	5.3
3	ITALY	94	7.0	84	10	10.6
4	UNITED KINGDOM	90	6.7	77	13	14.4
5	SPAIN	47	3.5	40	7	14.9
6	AUSTRALIA	39	2.9	33	6	15.4
7	JAPAN	38	2.8	36	2	5.3
8	GERMANY	33	2.4	22	11	33.3
9	INDIA	33	2.4	32	1	3.0
10	CANADA	30	2.2	28	2	6.7

**Figure 5:**
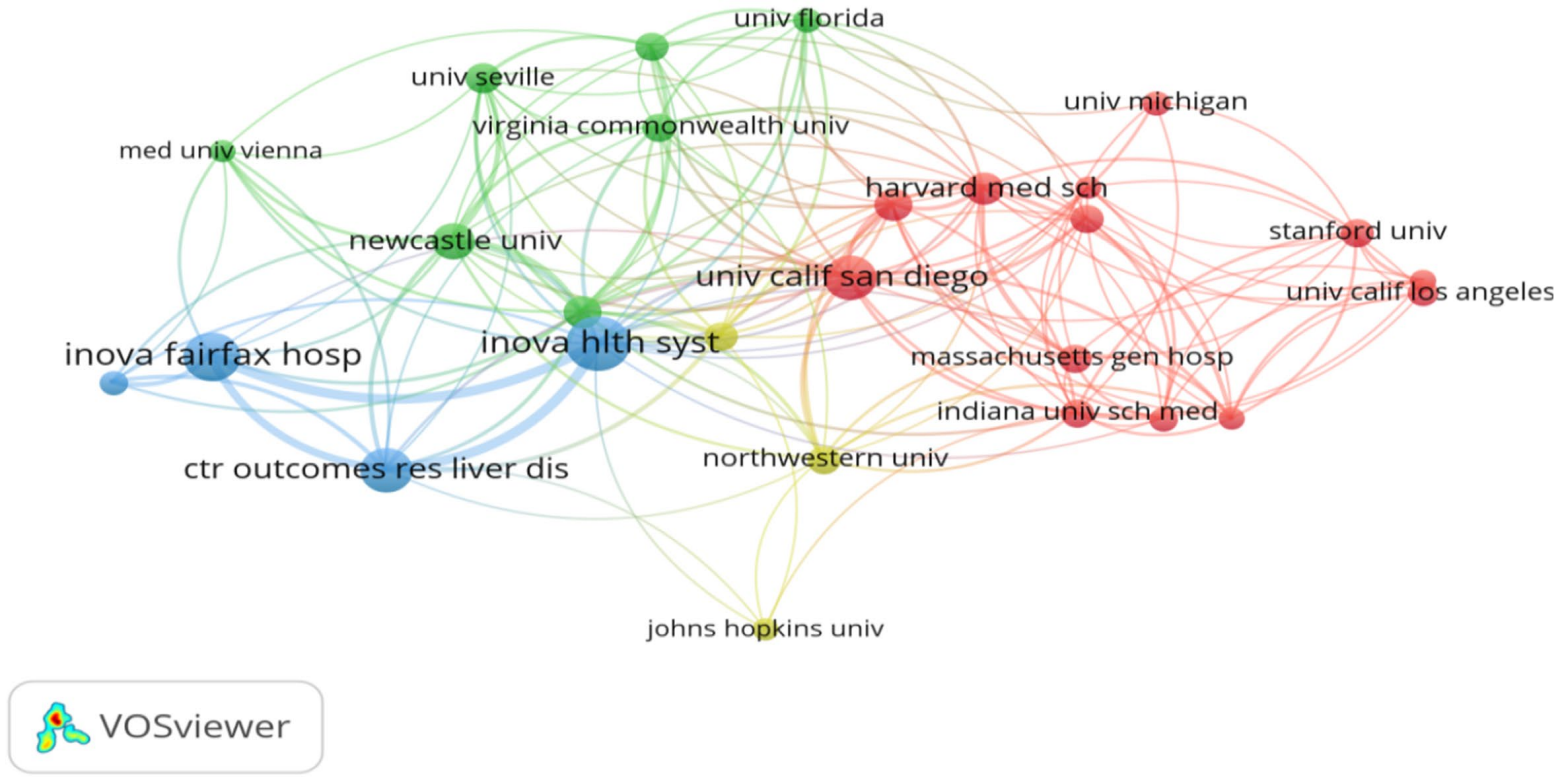
Clustering analysis of collaborative ties among institutions with over 10 publications.

Institutional leadership: Among the top 10 institutions with the highest number of publications (Table 3), University of California System (United States) leads with 67 contributions, closely followed by Harvard University (United States) with 54 publications and Inova Health System (United States) with 48 publications. Additionally, Figure 5 illustrates the collaboration network among the top 30 institutions, highlighting the strongest collaborative ties between Harvard University and Inova Health System, with a Total Link Strength (TLS) of 58.

**Table 3:** The top 10 institutions with the highest productivity.

Rank	Title of the institution	Literature	Nation
1	UNIVERSITY OF CALIFORNIA SYSTEM	67	The USA
2	HARVARD UNIVERSITY	54	The USA
3	INOVA HEALTH SYSTEM	48	The USA
4	UNIVERSITY OF BIRMINGHAM	47	The UK
5	UNIVERSITY OF CALIFORNIA SAN DIEGO	45	The USA
6	HARVARD MEDICAL SCHOOL	42	The USA
7	INOVA FAIRFAX HOSPITAL	42	The USA
8	MAYO CLINIC	41	The USA
9	UNIVERSITY OF BARCELONA	41	SPAIN
10	UNIVERSITY COLLEGE LONDON	40	The UK

### Scholarly ecosystem: periodicals and researchers

**Figure 6:**
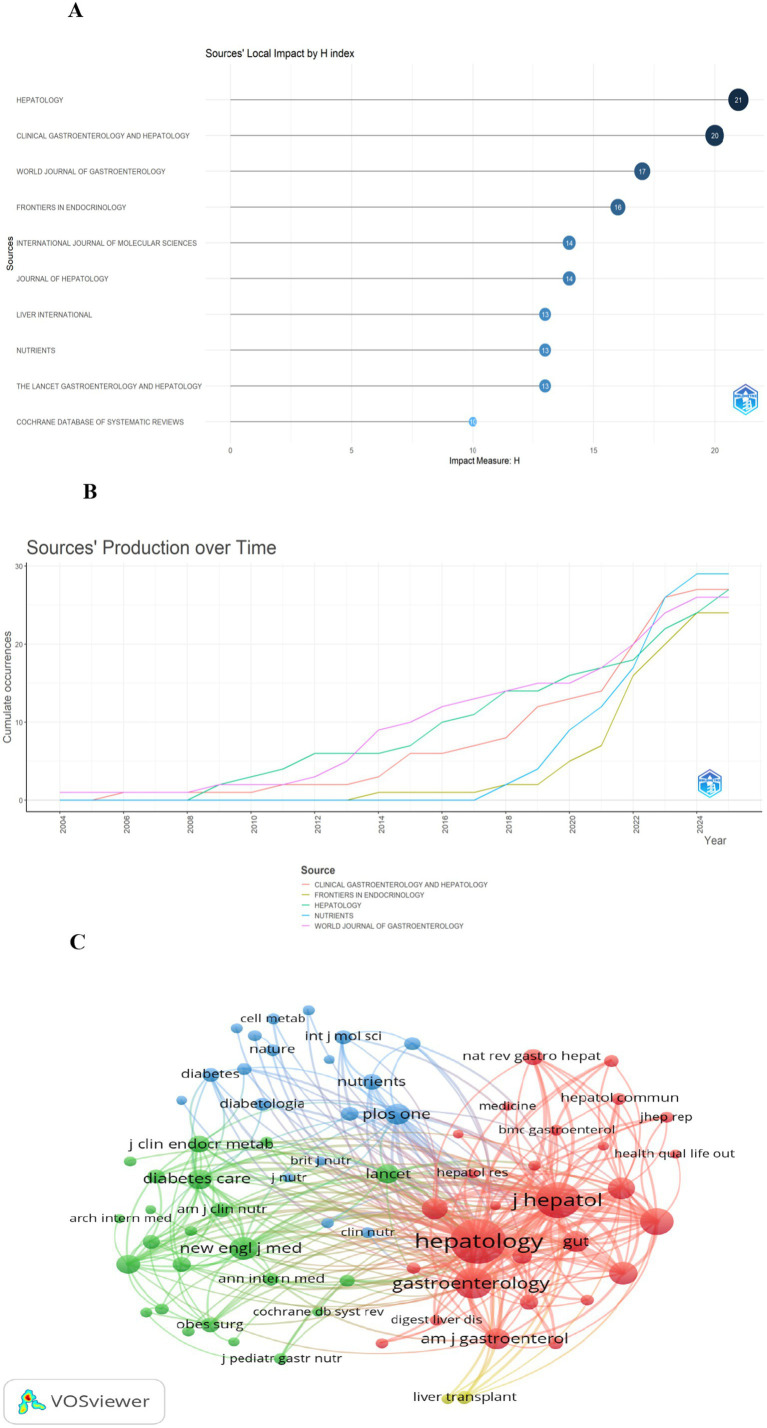
Overview of leading journals and co-citation analysis. Panel **(A)** is a ranking chart of journal H-indexes. Among them, the Journal of Hepatology has the highest H-index. Panel **(B)** shows the annual output trend of 5 core journals. Specifically, Nutrients has witnessed rapid growth in its publication volume since 2020. Panel **(C)** is a journal co-citation network. In this network, Hepatology, Journal of Hepatology, and Gastroenterology form the core co-citation cluster. The lines between them are the thickest, indicating their “pillar” status in the literature citation of this field.

Journal influence: A total of 1,347 articles from 617 journals were included in this study. The top 10 journals by publication volume, along with their most recent 2024 Impact Factors (IF), are presented in Table 4 (18). These journals comprise Nutrients (*n* = 29, IF = 5), Hepatology (*n* = 27, IF = 15.8), Clinical Gastroenterology and Hepatology (*n* = 27, IF = 12), World Journal of Gastroenterology (*n* = 26, IF = 5.4), and Frontiers in Endocrinology (*n* = 24, IF = 4.6). To assess the influence of these journals, the Bibliometrix R package was utilized, with measurements based on the H-index. The Journal of Hepatology had the highest H-index (Figure 6A) (22). Publication trends over the years are shown in Figure 6B. For co-citation analysis, VOSviewer was used to analyze source titles, including journals with at least 100 citations. Seventy-two journals were identified based on total link strength (Figure 6C). The top five journals with the highest total link strength were Hepatology, Journal of Hepatology, Gastroenterology, Clinical Gastroenterology and Hepatology, and The New England Journal of Medicine.

**Table 4:** The top 10 journals with the highest publication output.

Rank	Periodical	Publication counts	Citation counts	Impact factor	Quartile ranking
1	NUTRIENTS	29	1,027	5	Q1
2	HEPATOLOGY	27	4,368	15.8	Q1
3	CLINICAL GASTROENTEROLOGY AND HEPATOLOGY	27	2,624	12	Q1
4	WORLD JOURNAL OF GASTROENTEROLOGY	26	1882	5.4	Q1
5	FRONTIERS IN ENDOCRINOLOGY	24	1,041	4.6	Q1
6	HEPATOLOGY COMMUNICATIONS	23	317	4.6	Q1
7	INTERNATIONAL JOURNAL OF MOLECULAR SCIENCES	21	1,565	4.9	Q1
8	LIVER INTERNATIONAL	20	731	5.2	Q1
9	PLOS ONE	17	314	2.6	Q2
10	JOURNAL OF HEPATOLOGY	15	4,744	33	Q1

**Figure 7:**
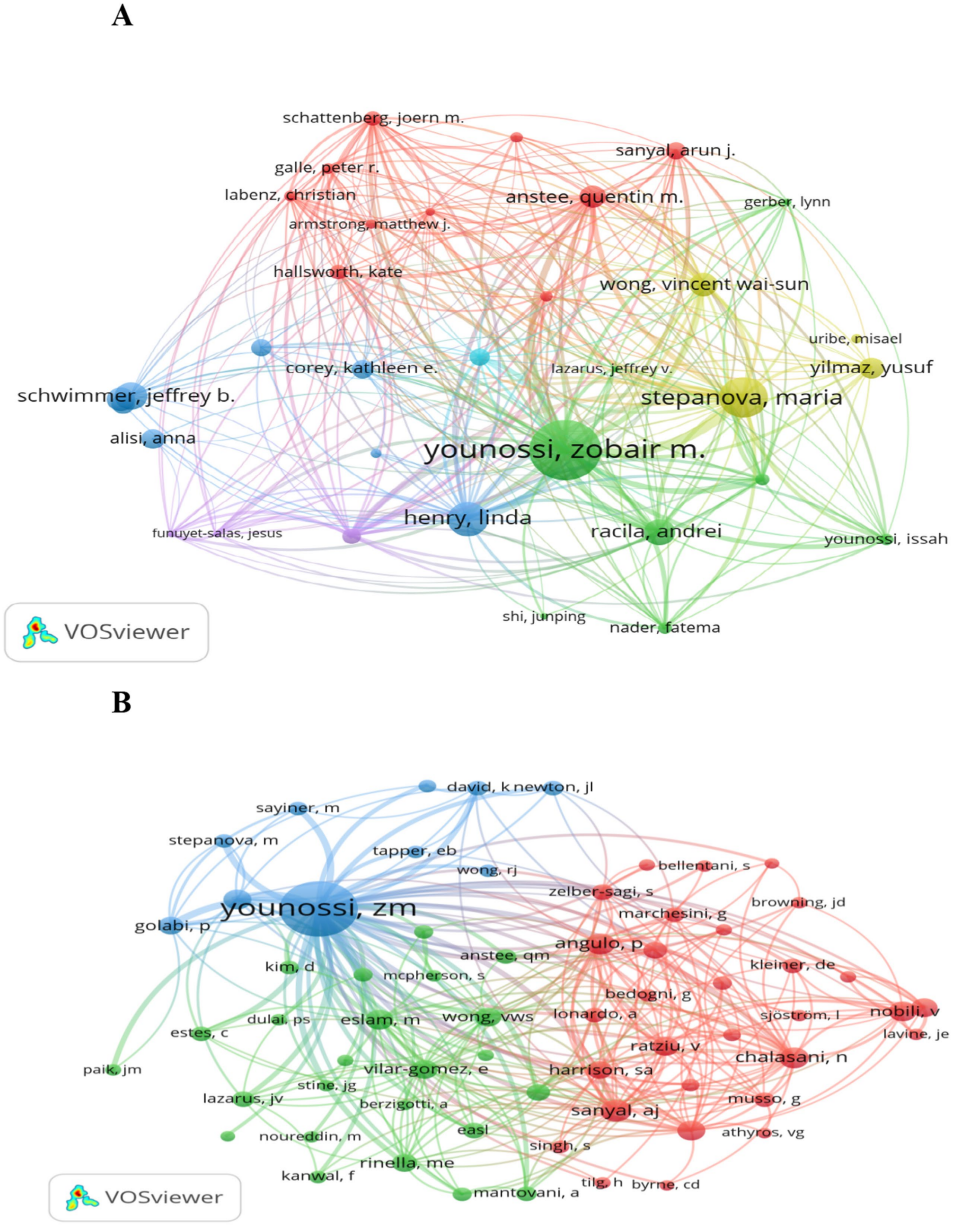
Depiction of cited and co-cited authors. **(A)** Cited author map: included here are authors cited in no fewer than 5 publications. Larger nodes signify greater citation frequency, while thicker lines indicate stronger collaborative connections in research on QOL of MAFLD patients. **(B)** Co-cited author co-occurrence map: this map highlights authors with over 40 co-citations. Node size and line thickness correspond to co-citation frequency and collaboration intensity, respectively. Node colors distinguish between distinct collaborative networks among co-cited authors.

Key authors and collaborative networks: Across the globe, 7,607 authors have contributed publications to this field. In this analysis, the top 15 authors—ranked by their publication counts—were designated as key authors. Table 5 provides detailed metrics, including h-index, g-index, and m-index, calculated over the 21-year period. Younossi Z leads in terms of publication quantity and has achieved the highest totals for citations, h-index, and g-index. Close collaborative ties among these key authors are visualized in Figure 7A. Additionally, Figure 7B highlights authors with over 100 co-citations, with the top three being Younossi Z (972 co-citations), Sanyal A (156 co-citations), and Wong V (141 co-citations).

**Table 5:** Publication and citation metrics for key authors.

Author	h_index	g_index	m_index	Total citation counts	Cumulative publications
YOUNOSSI Z	37	76	1.947	8,987	76
STEPANOVA M	20	31	1.333	3,025	31
WONG V	17	20	1.545	2,512	20
ANSTEE Q	16	20	1.778	2,398	20
SANYAL A	16	25	1.067	2,513	25
HENRY L	15	23	1.364	2,203	23
SCHWIMMER J	15	17	0.714	2,936	17
SCHATTENBERG J	14	23	2	1955	23
GOLABI P	12	13	1.2	1,302	13
ZELBER-SAGI S	12	12	0.923	2,283	12
LOOMBA R	11	18	0.647	1,121	18
ARMSTRONG M	10	12	0.667	472	12
CUSI K	10	14	1	1,300	14
NOUREDDIN M	10	12	2	1,107	12
RACILA A	10	11	1	1,323	11

Keywords and Research Frontiers Keyword Standardization: A thesaurus was employed (Table 6) to enable more precise counting of keyword occurrence frequencies.

**Table 6:** Term thesaurus.

Denomination	Replace by
Quality-of-life	Quality of life
Health-related quality of life	Quality of life
NAFLD	MAFLD
Fatty liver-disease	MAFLD
Non-alcoholic steatohepatitis	MAFLD
Non-alcoholic fatty liver disease	MAFLD
Non-alcoholic fatty liver disease	MAFLD
Non-alcoholic fatty liver	MAFLD
Non-alcoholic steatohepatitis	MAFLD
Non-alcoholic fatty liver disease (NAFLD)	MAFLD
Steatohepatitis	MAFLD
Fatty liver	MAFLD

**Figure 8:**
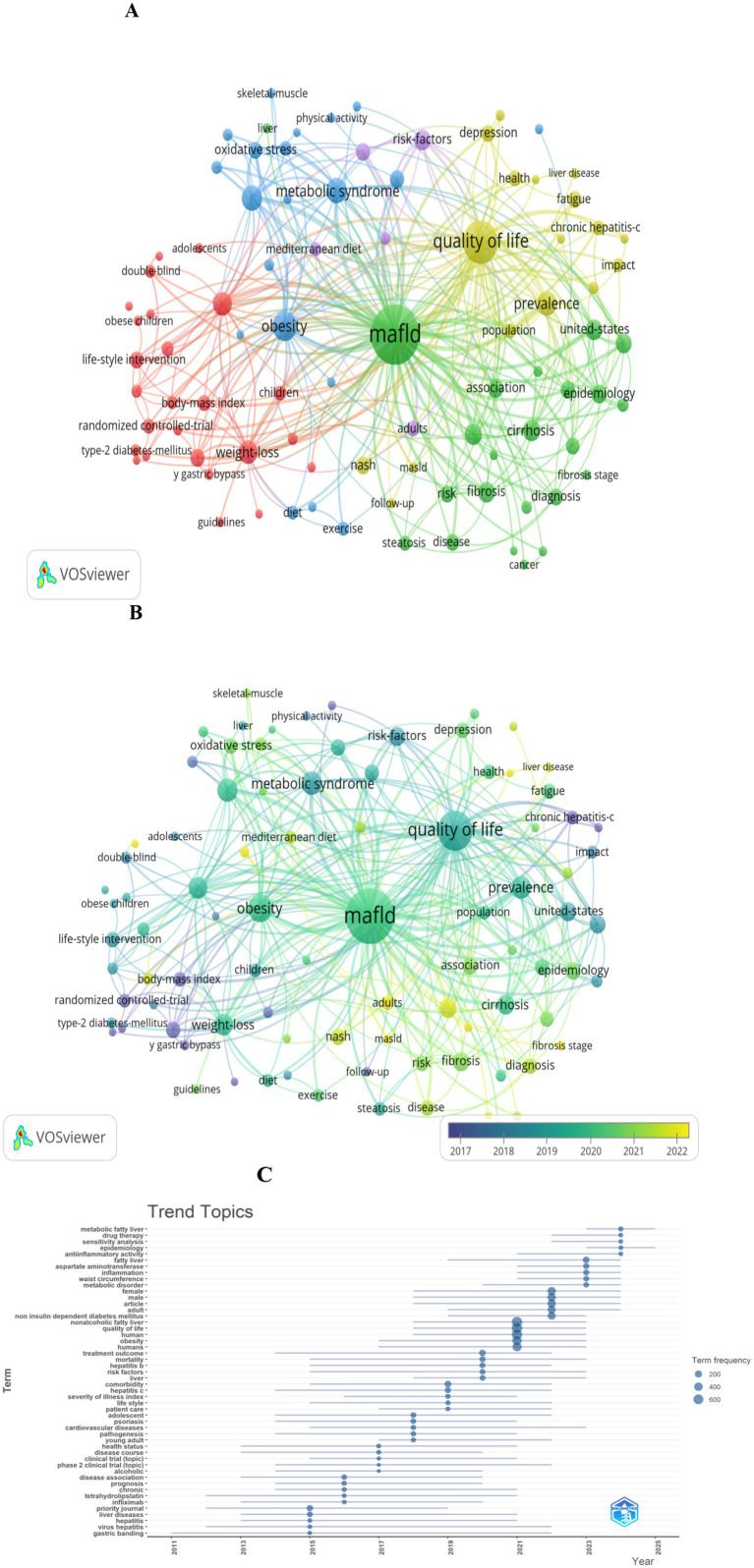
Keyword analysis in literature on the QOL of MAFLD patients. **(A)** Visualization of the keyword co-occurrence network associated with the QOL of MAFLD patients. Keywords are grouped into five clusters by color, with larger nodes indicating more frequently occurring terms. **(B)** Keywords shaded by their average appearance time: blue denotes the early phase, while yellow signifies the later phase. **(C)** Topic distribution graph centered on the QOL of MAFLD patients.

Clusters of Keyword Co-occurrence: Figures 8A,B provide visualizations of 90 keywords that meet a co-occurrence threshold of ≥10 within the network. The top 10 keywords were: MAFLD (*n* = 372), quality of life (*n* = 196), obesity (*n* = 93), metabolic syndrome (*n* = 71), insulin-resistance (*n* = 67), prevalence (*n* = 66), weight-loss (*n* = 61), hepatic steatosis (*n* = 59), management (*n* = 50), and cirrhosis (*n* = 50).

Five colors represent distinct keyword clusters: The red cluster centers on obesity management, including terms such as weight-loss, body-mass index, and bariatric surgery; the blue cluster centers on metabolic health, including metabolic syndrome, obesity, insulin-resistance, and oxidative stress; the green cluster centers on MAFLD-related pathology and epidemiology, including cirrhosis, fibrosis, epidemiology, and diagnosis; the yellow cluster centers on health impacts, including quality of life, depression, fatigue, and liver disease; the purple cluster center on risk factors, including cardiovascular-disease, mediterranean diet, and life-style.

Emerging Research Frontiers: In terms of research trends, key themes within this field (Figure 8C) that have emerged include drug therapy, sensitivity analysis, epidemiology, anti-inflammatory activity, aspartate aminotransferase, among others.

## Discussion

### Research growth and drivers

The 18.76% annual growth rate reflects increasing recognition of QOL as a critical outcome in MAFLD management. The accelerated expansion post-2018 aligns with the reclassification of NAFLD to MAFLD, emphasizing metabolic dysfunction as a core pathogenesis (23–25), as well as the publication of guidelines integrating QOL assessment into clinical care (26–28). Ultimately, this growth underscores the shift from a purely hepatocentric focus to holistic patient-centered outcomes.

### Geographic and institutional leadership

The United States’ dominance (27.3%) stems from robust funding for metabolic disease research, well-established networks integrating hepatology, academia, industry and public health, as well as its leadership in technological innovation and setting international standards (29, 30). China’s emergence (12.7%) reflects its strategic investments driven by the rising disease burden, incorporation into the “Healthy China” framework, advancements in technology, and integration of interdisciplinary collaboration, as well as large-scale epidemiological studies targeting the Chinese population (31–33). Additionally, strong international collaborations highlight the global nature of MAFLD as a public health challenge, with partnerships between high-income countries and regions with rising MAFLD burdens facilitating context-specific research (5, 34).

Leading institutions such as the University of California and Harvard University excel in translational research, bridging basic science on metabolic pathways and clinical interventions to improve QOL for patients. Their collaborations with clinical centers (e.g., Inova Health System) consistently demonstrate the value of academic-clinical partnerships in advancing patient-centered outcomes (35, 36).

### Journal and scholarly leadership

Hepatology journals such as Hepatology and Journal of Hepatology play a foundational role, leading in publication volume, H-index, and co-citation link strength. Meanwhile, the prominence of Nutrients (*n* = 29) and Frontiers in Endocrinology (*n* = 24) clearly indicates a shift from liver-centric research to integration with nutrition and endocrinology, aligning with the reclassification of NAFLD to MAFLD that emphasizes metabolic dysfunction. High-impact journals such as The New England Journal of Medicine within co-citation networks further confirm the field’s relevance to broader clinical discourse.

Zobair M. Younossi stands as the preeminent scholar in research on QOL in MAFLD, with contributions that span academic leadership, clinical innovation, and global advocacy. He has published over 800 papers on MAFLD and NASH. Notably, his pivotal leadership in renaming NAFLD to MAFLD—emphasizing metabolic dysfunction as a core driver—aligned with and accelerated the field’s shift toward holistic, patient-centered inquiry (37).

### Analysis of keywords and research frontiers

The QOL in MAFLD patients is a multifaceted construct shaped by complex interactions between disease-specific characteristics, metabolic comorbidities, and psychosocial factors (38, 39). Against this backdrop, this analysis identifies core keywords related to determinants of QOL in MAFLD, synthesizes current insights into how they interrelate, evaluates emerging interventions, and highlights key research frontiers to guide future inquiry.

#### Determinants of QOL in MAFLD

MAFLD-related QOL is not solely dictated by liver pathology but arises from the complex interplay of physiological, metabolic, and psychosocial stressors, underscoring the need for holistic assessment approaches.

##### Disease-specific characteristics

Disease-specific characteristics directly impact QOL through both overt and subtle mechanisms. Hepatic steatosis, inflammation, and fibrosis correlate inversely with QOL, with advanced fibrosis and cirrhosis driving substantial declines in physical function and emotional well-being—often exceeding 30% in QOL declines due to complications like esophageal varices and hepatic encephalopathy (40). Notably, approximately 80% of MAFLD patients exhibit no significant elevation in liver enzymes (41), yet subclinical manifestations (e.g., fatigue, sleep disturbances) arising from lipotoxicity and insulin resistance indirectly erode QOL over time (42). Thus, this “occult damage” highlights the limitations of relying solely on biochemical markers to gauge patient-reported outcomes.

##### Metabolic comorbidities

Metabolic comorbidities—including obesity, type 2 diabetes mellitus (T2DM), and metabolic syndrome (MetS)—exacerbate QOL impairment through bidirectional interactions with MAFLD. Obese patients, particularly those with central or sarcopenic obesity, experience heightened somatic symptoms (e.g., abdominal fullness, reduced exercise tolerance), with sarcopenic obesity associated with 1.5-fold greater QOL deficits due to muscle dysfunction (43). T2DM, present in 28.3% of MAFLD patients, overlaps symptomatically with MAFLD (e.g., fatigue, peripheral neuropathy), impairing daily functioning, while glycemic fluctuations further drive hepatic fat deposition (44–46). Similarly, MetS triples the risk of QOL reduction, as hypertension-related dizziness and dyslipidemia-induced fatigue compound liver-related symptoms to limit work capacity and social participation (47, 48).

##### Psychosocial factors

Psychosocial factors represent critical, often underappreciated determinants. MAFLD patients exhibit elevated rates of depression and anxiety, linked to fear of disease progression, stigma, and uncertainty about prognosis (49, 50). NAFLD imposes significant socioeconomic burdens: its healthcare costs are nearly double those of age-matched non-affected populations, while the lack of specific drug therapies and cost disparities in interventions exacerbate access inequalities, perpetuating a cycle of disease burden and economic strain (30, 51).

#### Interventions to improve QOL

Addressing MAFLD-related QOL requires multidimensional interventions that target metabolic, hepatic, and psychosocial pathways simultaneously.

##### Lifestyle modifications

Lifestyle modifications remain foundational. Specifically, calorie restriction and structured diets (e.g., Mediterranean diet, low-glycemic index diet) reduce hepatic fat and alleviate MAFLD-associated symptoms like abdominal distension, while intermittent fasting enhances exercise tolerance (52–54). Aerobic exercise improves insulin resistance and reduces fatigue levels, and resistance training boosts muscle mass in sarcopenic obesity, increasing activity capacity (55–57). These interventions highlight the transformative potential of non-pharmacological approaches to disrupt the “metabolism-liver” axis.

##### Psychological support and social interventions

Psychological support and social interventions jointly alleviate emotional burdens. Among them, cognitive-behavioral therapy (CBT) effectively reduces anxiety and depression, thereby improving emotional functioning and strengthening social participation (58). Community-based initiatives—such as patient support groups, subsidized healthy food programs, and insurance coverage expansions—alleviate economic stress and improve the QOL for vulnerable populations (59).

##### Multidisciplinary team (MDT) management

Multidisciplinary team (MDT) management optimizes care for complex comorbidities. Collaboration between gastroenterology, endocrinology, cardiology, and psychology ensures synchronized control of T2DM, hypertension, and other related conditions (60). This model reflects the systemic nature of MAFLD and its impact on QOL.

#### Pharmacological advances

Drugs such as GLP-1 class medications, Resmetirom, and Chiglitazar are current research hotspots, but most of them still require larger-scale and long-term clinical trials to verify their efficacy and safety. Future treatments may tend to use multi-target combination therapy to achieve more comprehensive metabolic regulation and liver protection.

##### Incretin-based drugs

GLP-1 receptor agonists, such as semaglutide, reduce hepatic fat deposition and improve insulin resistance, with studies demonstrating benefits for liver fat content and inflammatory markers in MASLD patients, though further research is needed to confirm long-term efficacy and antifibrotic effects (61). GLP-1/GIP dual receptor agonists like tirzepatide have shown potential in improving hepatic steatosis and fibrosis in clinical trials, with particularly significant effects in patients with moderate to severe fibrosis (62).

##### Thyroid hormone receptor β agonists

Resmetirom is the first drug approved by the US FDA for the treatment of MASLD patients with moderate to severe liver fibrosis (stage F2–F3). It can improve hepatic steatosis, inflammation, and fibrosis, but it is relatively expensive and requires long-term administration (63).

##### Peroxisome proliferator-activated receptor (PPAR) agonists

Chiglitazar, the world’s first full PPAR agonist, can simultaneously activate the three subtypes PPARα, γ, and δ. It significantly reduces liver fat content, improves liver function and metabolic indicators, and exhibits good safety (64).

Systematically summarized interventions are presented in Table 7.

**Table 7:** Summary of main interventions to improve QOL in MAFLD patients.

Intervention type	Representative studies (references)	Core measures	QOL-related outcome indicators
Lifestyle Intervention	(52, 55, 57)	Mediterranean diet, aerobic exercise, intermittent fasting	Reduce hepatic fat; Increase in physical function scores; Reduction in fatigue symptoms
Psychological Intervention	(58)	Cognitive Behavioral Therapy (CBT)	Decrease in anxiety/depression scale scores; Improved social participation
Multidisciplinary Team (MDT) Management	(60)	Collaboration among gastroenterology, endocrinology, and psychology departments	Increase in metabolic indicator control rate; Improvement in comprehensive QOL scores
Pharmacological Intervention (GLP-1 Receptor Agonists)	(61, 62)	Semaglutide, Tirzepatide	Reduction in liver fat content; Improvement in physical function scores
Pharmacological Intervention (Thyroid Hormone Receptor β Agonists)	(63)	Resmetirom	Improvement in liver fibrosis; Reduction in fatigue scores
Pharmacological Intervention (PPAR Pan-Agonists)	(64)	Chiglitazar	Remission rate of metabolic syndrome; Improvement in QOL emotional dimension scores

#### Research frontiers and future directions

##### Long-term impact of early intervention

Studies indicate that lifestyle or pharmacological interventions during simple steatosis are associated with 25–30% higher QOL scores over 10 years compared with late intervention, emphasizing the need for prospective cohort data to confirm durability (27, 65).

##### Mechanistic insights

Targeting insulin resistance (e.g., via PI3K/Akt pathways) (66) and modulating the gut-liver axis (e.g., with probiotics) (67) may simultaneously improve hepatic and metabolic symptoms, offering novel QOL-enhancing targets.

##### Individualized care

Genomic (e.g., PNPLA3 polymorphism) (68) and phenotypic (e.g., sarcopenic vs. central obesity phenotypes) (69) stratification could optimize treatment selection, improving response rates and minimizing side effects.

##### Policy and public health

Integrating MAFLD into chronic disease frameworks, expanding screening in underserved populations, and combating liver disease stigma through education could reduce disparities in QOL (70–72).

### Limitations

This study only incorporated literature from the WoSCC and Scopus databases. These two databases are widely recognized in bibliometric research. However, they might still omit regional studies published in other databases like PubMed Central and Embase, for example, non-English journal articles and local studies from developing countries. As a result, a slight “Euro-American bias” exists in the global research landscape. Moreover, some gray literature, such as conference abstracts and dissertations, was not included, which could lead to an underestimation of the early exploration of emerging research directions.

The study spans a period from 2004 to 2025. However, when the analysis was carried out, the data for 2025 was incomplete. This might cause a slight distortion in the calculation of annual trends. Nevertheless, despite the provisional nature of the 2025 data, the fact that there were a significant number of articles in the first 5 months of 2025 and that there is a predicted upward trend suggests that the current data, even though it’s incomplete, still has value and can offer meaningful insights for our research.

## Conclusion

MAFLD-related QOL is a dynamic outcome that is shaped by disease progression, metabolic comorbidities, and psychosocial contexts. Effective management of this outcome requires integrated strategies. These strategies encompass lifestyle interventions, psychological support, MDT care, and precision pharmacology, and they should be tailored to individual patient needs. Future research in this field must prioritize three key areas: the collection of long-term data, the achievement of mechanistic clarity, and the development of equitable interventions. The ultimate goal is to help patients with MAFLD achieve holistic “physical-psychological-social” well-being.

## Data Availability

The original contributions presented in the study are included in the article/supplementary material, further inquiries can be directed to the corresponding author.
